# Transarterial chemoembolization (TACE) combined with sorafenib versus TACE for hepatocellular carcinoma with portal vein tumor thrombus: a systematic review and meta-analysis

**DOI:** 10.18632/oncotarget.15075

**Published:** 2017-02-03

**Authors:** XiuPing Zhang, Kang Wang, Meng Wang, Guang Yang, XiaoFei Ye, MengChao Wu, ShuQun Cheng

**Affiliations:** ^1^ Department of Hepatic Surgery VI, Eastern Hepatobiliary Surgery Hospital, Second Military Medical University, Shanghai, China; ^2^ Department of Medical Statistical, Second Military Medical University, Shanghai, China; ^3^ Company 5 of Student Brigade, Second Military Medical University, Shanghai, China

**Keywords:** hepatocellular carcinoma, PVTT, TACE, sorafenib, combined treatment

## Abstract

**Background:**

The benefits of transarterial chemoembolization plus sorafenib (TACE-S) in hepatocellular carcinoma (HCC) with portal vein tumor thrombus (PVTT) remain controversial. We compared the effectiveness and safety of TACE-S and TACE for HCC with PVTT.

**Methods:**

The Cochrane Library, PubMed, EMBASE, Chinese National Knowledge Infrastructure, VIP, Wan Fang, and Sino Med databases were systematically searched for studies of HCC with PVTT treated using TACE-S. Two authors independently extracted study outcomes, including overall survival (OS), time to progression (TTP), objective response (tumor response) and adverse events (AEs).

**Results:**

Eight high-quality, retrospective studies with 1091 patients (TACE-S=356, TACE=735) were included in the review. Five retrospective studies with 973 patients (TACE-S=238, TACE=735) were included in the meta-analysis. The objective response rate (ORR, OR=3.59, 95% CI=1.74–7.39; I^2^=21%, *P*=0.0005) and disease control rate (DCR, OR=4.72, 95% CI=1.75–12.72; I^2^=56%, *P*=0.002) favored TACE-S. TACE-S significantly increased 6-month OS (OR=3.47; 95% CI=2.47–4.89; I^2^=0%, *P* < 0.00001) and 1-year OS (OR=3.10; 95% CI=2.22–4.33; I^2^=41%, *P* < 0.00001). The hazard ratio (HR) for OS (HR=0.62; 95% CI=0.51–0.75; I^2^=30%, *P* < 0.00001) also indicated that TACE-S was superior to TACE. TACE-S with PVTT had better outcomes in the first-order portal vein branch and lower-order portal vein branches than in the main portal vein and upper branches to superior mesenteric vein. The most common AEs were hand-foot skin reaction (HFSR, 178; 73%), diarrhea (142; 58%) and alopecia (76; 31%); AEs of grade 3/4 were rare.

**Conclusions:**

TACE-S may improve OS, ORR, TTP and DCR for HCC patients with PVTT compared to TACE.

## INTRODUCTION

Hepatocellular carcinoma (HCC) is the most common cancer and has dismal outcomes [1, 2]. Portal vein tumor thrombosis (PVTT) is the most commonly recognized risk factor for prognosis. PVTT occurs in 44-62.2% of patients with advanced HCC and is associated with a natural median survival time (MST) of 2.7-4 months [3, 4]. Despite recent advances in the treatment of such patients, the treatment strategies for patients with HCC with PVTT remain controversial.

According to the Barcelona Clinic Liver Cancer (BCLC) treatment strate gy, sorafenib is the only recommended treatment for patients with HCC with PVTT. However, recent studies have shown that TACE can be safely performed even in HCC patients with PVTT if they have good liver function and sufficient collateral circulation after portal vein occlusion [5, 6]. Some recent studies have suggested that TACE might benefit PVTT, but its effect was limited [7–9]. TACE-S appears to be a promising method for HCC patients with PVTT. Several retrospective and prospective studies of this therapy have summarized the efficacy and safety for PVTT patients [10, 11]. Moreover, some meta-analyses of this new treatment for unresectable HCC without PVTT have optimized this treatment modality [12–15]. However, additional randomized, controlled studies (RCTs) of TACE-S for HCC with PVTT are needed to confirm the efficacy and safety of this method.

Although this combination therapy has been used in patients suffering from HCC with PVTT, the current data on therapeutic effects are controversial, and its clinical role has not been decided. Here, we performed the first systematic review and meta-analysis of clinical trials to assess the efficacy and safety of TACE-S and TACE therapy alone for HCC patients with PVTT.

## RESULTS

### Patient characteristics

**Figure 1:**
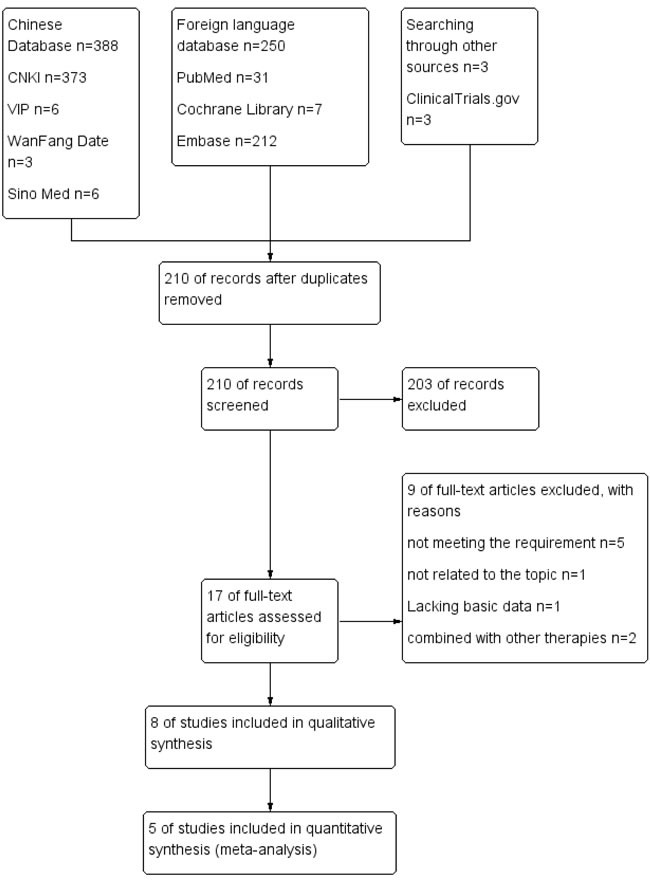
PRISMA flow diagram of the process for the identification of eligible studies CNKI: Chinese National Knowledge Infrastructure; VIP: Chongqing VIP database for Chinese Technical Periodicals; Wan Fang: Wan Fang Database; Sino Med: Chinese Biological Medical Literature Database.

The search strategy identified 1279 relevant studies, of which 1069 were duplicates. A total of 203 references were excluded after the titles and abstracts were screened; then, 9 studies were excluded for other reasons, as shown in Figure 1. Finally, 8 retrospective, controlled studies [7, 10, 20–25] were eligible for inclusion and qualitative synthesis, and 5 studies were included in the meta-analysis (Figure 1A). This systematic review included a total of 8 retrospective studies. Table 1 presents the basic characteristics of the included studies. A total of 1091 patients with HCC and PVTT were included, of which 356 received TACE-S therapy and 735 received TACE alone. More men than women with HCC and PVTT were included in the analysis. The tumor size mostly centralized on 5-10 cm. The age of patients ranged from 40 to 70 approximately. The baseline liver function of most of the participants was Child-Pugh A [10, 21, 23–25]. There was no difference between TACE-S and TACE group in patients’ liver function. The baseline ECOG of the included patients was reported in six studies, and the proportion of patients without considerable ECOG varied among the studies. The serum AFP level of these patients was more than 400 mg/L according to six studies. Five studies reported virology, and most of the participants had HBV. The baseline characteristics of the patients in the studies were recorded (Table 1).

**Table 1:** Patient characteristics

Study (year)	Study design	Treatment	Country	Patients	Type of PVTT(III/II/I)	Tumor size(cm) (<5/5-10/>10)	AGE	Sex(M/F)	Child-Pugh (A/B/C)	ECOG Ps(0/1/2)	Virology HBV/HCV/Other	Total bilirubin level (μmol/L)	Serum albumin level (g/L)	AFP(mg/L)(≦400/ >400)
**Hao 2015**	R	T+S	China	32	10/14/8^a^	NA	45.6±12.1	29/3	NA	14/18-(0/1-2)	NA	NA	NA	13/19
		T		38	11/21/6^a^	NA	48.3±9.8	34/4	NA	15/23-(0/1-2) ^c^	NA	NA	NA	15/23
**Chen 2014**	R	T+S	China	21	10/7/4	6/10/5	57±14	17/4	20/1/0	4/12/5	NA	NA	NA	NA
		T		23	7/13/3	5/9/9	50±12	19/4	20/3/0	4/11/8	NA	NA	NA	NA
**Luo 2014**	R	T+S	China	26	21/15/15^b^	NA	45.78±11.3	30/21	13/19/19 ^b^	NA	NA	NA	NA	NA
		T		25		NA				NA	NA	NA	NA	NA
**Zhu 2014**	R	T+S	China	46	10/19/17	NA	48.46±8.1	39/7	39/7/0	22/24-(0/1-2) ^c^	38/5/3	32.1± 9.7	34.6±4.5	23/23
		T		45	11/21/13	NA	51.96±12.2	38/7	39/6/0	20/25-(0/1-2) ^c^	40/1/4	33.8±11.2	35.2±4.3	19/26
**Zhang 2015**	R	T+S	China	45	NA	NA	50.1±8.8	43/2	21/13/11	0/41/4	44/1/0	36.8±5.7	20.4± 6.8	3/42
**Pan 2014**	R	T+S	China	41	33/8/0 ^a^	0/25/16	52(range:29-73)	38/3	35/6/0	20/21/0	40/1/0	26/15(≦2/>2)	5/36(<35/>35)	22/19
**Chen JW 2013**	R	T+S	China	32	10/10/12	NA	44	29/3	22/10/0	2/28/2	32/0/0	NA	NA	4/18
**Wang 2016**	R	T+S	China	113	37/45/31	29/84/0	58/55(≦50/>50)	77/36	110/3/0	NA	29/0/0	49/64(≦18.8/ >18.8)mmol/L	39/74(≦34/ >34)	45/68
		T		604	269/288/47	0/25/16	285/319	534/70	567/37/0	NA	125/0/0	353/251	130/474	230/374

Note: R: Retrospective study, NA: Not applicable, TACE: Transarterial chemoembolization, S: sorafenib, OG PS: eastern Cooperative Oncology Group Performance Status, Type A: Main portal vein, Type B: First-order portal vein branch, Type C: Second- or lower-order portal vein branches. AFP: alpha fetoprotein

a: The type of PVTT in study Hao2015 and Pan2014 was performed by Cheng's classification [28]. The data of PVTT type in Cheng's classification were that 1/2/3/4 Hao2015 T+S: 8/14/7/3, T: 6/21/7/4, Pan2014 0/8/23/10. We change type I by Cheng's classification to type C, type II by Cheng's classification to type B, type III by Cheng's classification to type A.

b: The both patient accepted combine treat and TACE alone are 21/15/15.

c: The study record both level1 plus level 2 number.

### Treatment regimens

The number of treatment cycles of TACE ranged from 1 to 8 times, and the mean number ranged from 2.4 to 3.6. The chemotherapeutic agents differed among the included studies. However, the embolic agents were same, such as lipiodol, gelatin sponge and Gelfoam The daily dosage of sorafenib was 400 mg bid in all studies. In most of the included studies, the time of use of sorafenib ranged from 1 to 7 days after the first TACE session, with no breaks before or after repeated TACE if adverse events of grade 3/4 did not occur. None of the patients received other treatments (Table 2).

**Table 2:** Procedures of TACE and sorafenib combination therapy

Study	TACE			Sorafenib
	Duration and interval	Chemotherapeutic agents	Embolic agents	
**Hao 2015**	Mean 2.4times (range: 1–5) 4–8 week interval	pirarubicin 30~60mg oxaliplatin 100~150mg	lipiodol 5~15ml	400mg bid at 3–7 days after the first TACE session, no breaks before or after repeated TACE
**Chen 2014**	2.5±1.1times	epirubicin, platinum agent, camptothecin	lipiodol, absorbable gelatin sponge and polyvinyl alcohol	400 mg bid at 7 days before the first TACE session and 14 days after it
**Luo 2014**	NA	10-40 mg pirarubicin 40-80 mg nedaplatin 500-1000 mg 5-FU	10-40ml mixed lipiodol	400 mg bid
**Zhu 2014**	Mean 3.6times (range1–8) 4–6 week interval	20–60 mg doxorubicin 20-50 mg lobaplatin	2–20 mL lipiodol 300–1000-μm polyvinyl alcohol particles	400 mg bid at 3–5 days after the first TACE session, no breaks before or after repeated TACE
**Zhang 2015**	Mean 2.6 (range: 1–5)	20–40mg epirubicin	10–20 mL lipiodol Gelfoam	400 mg bid after 1–3 days TACE, and administration was suspended on the day a repeated TACE procedure was performed
**Pan 2014**	NA	40-60 mg epirubicin 6-10 mg mitomycin C	8-30 ml lipiodol gelatin sponge	400 mg bid after 3 days TACE A 3-day interruption in sorafenib was adopted after each subsequent TACE cycle.
**Chen JW 2013**	NA	50 mg lobaplatin 30 mg THP 1.0g 5-FU	super liquefied iodized oil, gelatin sponge and polyvinyl alcohol	400 mg bid after 3–7 days TACE
**Wang 2016**	6 to 8 weeks interval	20 to 60mg doxorubicin hydrochloride, 5mg cisplatin	5 to 30 ml lipiodol Gelfoam fragments	400mg bid twice daily at 1 week after the first TACE session

### Tumor response

The seven of the included eight studies reported response assessment by RECIST (Response Evaluation Criteria in Solid Tumors), and the outcomes are shown in Table 3. The tumor response to treatment was classified as complete response (CR), partial response (PR), stable disease (SD), and progression of disease (PD). The DCR (DCR defined as CR + PR + SD) was reported in 7 studies and ranged from 4.8% to 80.5% in the combination group and 0% to 68.0% in the TACE-alone group. The ORR (ORR defined as CR + PR) ranged from 0% to 46.2% in the combination group and 0% to 32.0% in the TACE-alone group. Because the follow-up time ranged from 2 months to 1 year, the results obviously varied. In addition, some studies did not list follow-up time, although it was reported by Hao [20], Zhu *et al.* [23]. and Pan *et al.* [24]. The DCR of the combination treatment was superior to that of TACE alone in the studies reported by Hao [20], Chen *et al.* [21], Luo LZ and Luo D [22] and Zhu *et al.* [23]. The DCR of combination treatment in all seven studies ranged from 4.8% to 80.5%, and the majority of DCRs were higher than 50%.

**Table 3:** Tumor responses in patients of included studies

Study	Type of PVTT	Follow-up time	Complete response	Partial response	Stable disease	Progressive disease	Death	Objective response rate (%)	Disease control rate (%)
**Hao 2015**	T+S	NA	0	10	8	14	0	31.3	56.2
	T	NA	0	4	6	28	0	10.5	26.3
**Chen 2014**	T+S	6months 1year	0 0	0 0	10 1	6 4	5 16	0.0 0.0	47.6 4.8
	T	6months 1year	0 0	0 0	0 0	5 0	18 23	0.0 0.0	0.0 0.0
**Luo 2014**	T+S	9months	0	12	8	6	0	46.2	76.9
	T	9months	0	8	9	8	0	32.0	68.0
**Zhu 2014**	T+S	NA	0	13	13	20	0	28.3	57
	T	NA	0	2	4	39	0	4.4	13
**Zhang 2015**	T+S	3months	0	9	15	12	9	20.0	56
**Pan 2014**	T+S	NA	1	7	25	8	0	19.5	80.5
**Chen JW 2013**	T+S	2months	0	8	12	12	0	25.0	62.5
**Wang 2016**	T+S T	NA NA	NA NA	NA NA	NA NA	NA NA	NA NA	NA NA	NA NA

Note: All used RECIST, response evaluation in solid tumors.

The Meta analysis used Chen2014 6month data.

**Figure 2:**
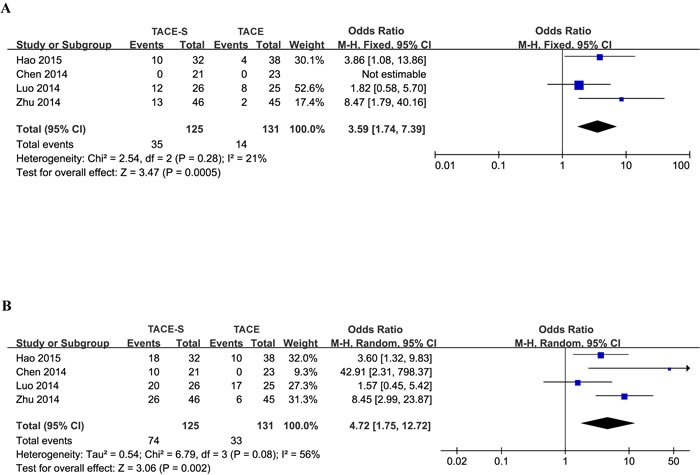
Forest plots for the comparison of tumor response in HCC patients with PVTT who received TACE-S or TACE alone Outcomes: **A**. ORR; **B**. DCR. The meta-analysis of ORR was performed using a fixed-effects model. The meta-analysis of DCR was performed using a random-effects model for significant heterogeneity.

Four studies [20–23] indicated that the DCR of combined treatment was superior to that of TACE alone. Meta-analysis of retrospective studies by ORR (OR=3.59, 95% CI =1.74-7.39; I2 = 21%, P=0.0005; Figure 2A) and DCR (OR=4.72, 95% CI =1.75-12.72; I2 = 56%, P=0.002; Figure 2B) suggested that patients who underwent the combination therapy tended to have better responses than those who underwent TACE treatment alone.

Significant heterogeneity in DCR was observed. Therefore, the effects were pooled using a random-effects model for between-study variability.

### Overall survival

The median OS was reported in seven of eight retrospective studies [7, 10, 20, 21, 23–25] (Table 4). Moreover, some data were calculated using survival curves. The median OS ranged from 7 to 13 months in the combination treatment group and 4 to 6.1 months in TACE-alone group. Six-month OS and 1-year OS were reported in six of eight retrospective studies [7, 20–24]. In the combination groups, 6-month OS ranged from 67.88% to 87.7%, and 1-year OS ranged from 23.9% to 65.1%. Moreover, 6-month OS ranged from 21.7% to 63.6%, and 1-year OS ranged from 0% to 36.8% in the TACE-alone treatment group [20–23].

**Table 4:** The outcomes of therapy for HCC with PVTT

Study (year)	Treatment	Patients	Median TTP (months)	Median OS(months)	Survival rate (%)
**Hao 2015**	T+S	32	NA	10.2	71.9(6months) 43.8(1year)
	T	38	NA	6.0	36.8(6months) 10.5(1year)
**Chen 2014**	T+S	21	6.2±0.5	8.4±1.1	76.2(6months) 23.9(1year)
	T	23	2.4±0.3	4.1±0.6	21.7(6months) 0 (1year)
**Luo 2014**	T+S	26	NA	NA	83.0(6months) 65.1(1year)
	T	25	NA	NA	63.6(6months) 36.8(1year)
**Zhu 2014**	T+S	46	6.0 (95%CI:4.9-7.1)	11.0 (95%CI:7.8-14.2)	82.6(6months) 45.7 (1year)
	T	45	3.0 (95% CI: 2.2-3.8)	6.0 (95% CI:4.9-7.1)	60.0 (6months) 17.8(1year)
**Zhang 2015**	T+S	45	3.0	7.0	NA
**Pan 2014**	T+S	41	7	13.0	87.7(6months) 53.6(1year)
**Chen JW 2013**	T+S	32	3	7	NA
**Wang 2016**	T+S	113	NA	8.92(95%CI:7.86-10.97)	67.88 (6months) 37.36 (1year)
	T	604	NA	4.79(95%CI:4.07-5.45)	41.56 (6months) 24.16 (1year)

**Figure 3:**
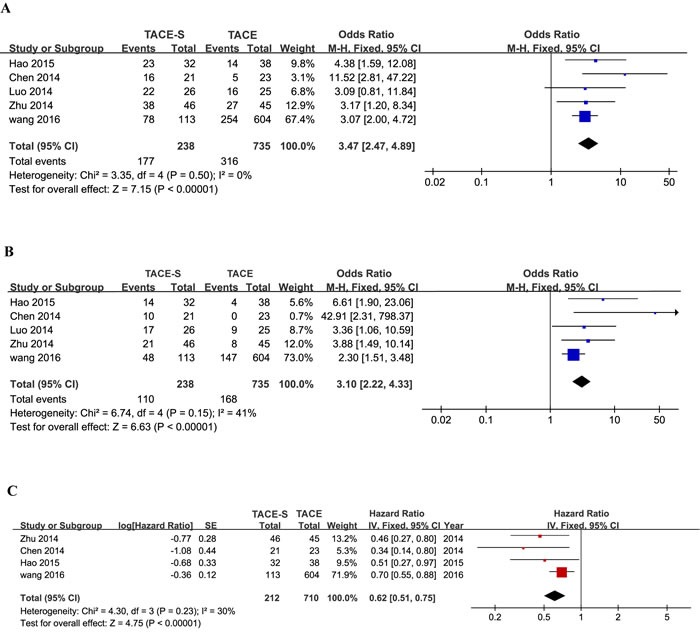
Forest plots for the comparison of odds ratios for overall survival in HCC patients with PVTT who received TACE-S or TACE alone Outcomes: **A**. 6-month overall survival; **B**. 1-year overall survival; **C**. the hazard ratio for overall survival. A fixed-effects model was used in the meta-analyses of the three outcomes.

Four studies [7, 20–23] with contrasting results were used to perform meta-analyses of 6-month OS, 1-year OS and HR for OS. Six-month OS in all available studies favored the combination group (OR=3.47; 95% CI=2.47-4.89; I^2^=0%, P<0.00001; Figure 3A), as did 1-year OS (OR=3.10; 95% CI=2.22-4.33; I^2^=41%, P<0.00001; Figure 3B). Due to this article written by Luo LZ and Luo D^22^ not providing the specific HR, 95% CI and survival curves, the study was excluded in a meta-analysis of HR. Similarly, meta-analysis of HR for OS suggested that the patients in the combination therapy group had significantly longer survival than those in the TACE therapy group (HR=0.62; 95% CI=0.51-0.75; I^2^=30%, P<0.00001; Figure 3C). The meta-analysis showed that OS was significantly greater in the TACE-S group than in the TACE-alone group.

### Time to progression

Five of eight retrospective cohort studies [10, 21, 23–25] reported the median TTP. The median TTP in the combination group ranged from 3 to 7 months in five studies [10, 21, 23–25]. The median TTP in the TACE-alone group was 2.4 and 3.0 months in two studies [21, 23]. Chen *et al*. [21] reported a median TTP of 6.2 months in the combination group versus 2.4 months in the TACE-alone group. Similarly, Zhu *et al*. [23] reported a median TTP of 6.0 months for TACE-S versus 3.0 months for TACE alone. The results suggested that combination therapy group had more time to progression than the TACE-alone group. Two studies [21, 23] compared different effects between TACE-S and TACE alone. Therefore, we did not conduct a meta-analysis (Table 4).

### Outcomes of patients with different types of PVTT

The 8 retrospective studies (Table 2) included a total of 1091 patients with HCC and PVTT, with 356 receiving TACE-S therapy. Some studies [22, 23, 25] analyzed outcomes using the Kaplan-Meier method according to the type of PVTT (PVTT in the main portal vein [type A], PVTT in the first-order portal vein branch [type B], and PVTT in the second- or lower-order portal vein branches [type C]), whereas other studies [20,24] defined PVTT or PVTT type using the Cheng's classification (Type I: tumor thrombus involving segmental branches of the portal vein or above; Type II: tumor thrombus involving the right/left portal vein; Type III: tumor thrombus involving the main portal vein trunk; Type IV: tumor thrombus involving the superior mesenteric vein) [26]. Compared with two classifications, type A was same to Type III; type B was similar to Type II; and type C was analogous to Type I. This article unified used Cheng's classification. In included studies, Type II was common in all different type of PVTT. All data indicated that patients undergoing combination therapy whose PVTT was in the first-order portal vein branch or lower-order portal vein branches, Type I-II, had better results than those whose PVTT was in the main portal vein or the upper branches to superior mesenteric vein, Type III-IV. (Table 5).

**Table 5:** The outcomes of patients with TACE plus sorafenib combination therapy for various PVTT types

Study (year)	Treatment	Type of PVTT	Patients	Median TTP(months)	Median OS(months)	DCR (%)
**Hao 2015**	T+S	Type I	8	NA	19.8	NA
		Type II	14	NA	10.3	NA
		Type III	7	NA	8.1	NA
		Type IV	3	NA	2.1	NA
	T	Type I	6	NA	10.2	NA
		Type II	21	NA	6.0	NA
		Type III	7	NA	3.0	NA
		Type IV	4	NA	2.0	NA
**Chen 2014**	NA	NA	NA	NA	NA	NA
**Luo 2014**	T+S	Type III	NA	NA	3	NA
	T+S	Type II	NA	NA	12	NA
	T+S	Type I	NA	NA	14	NA
	T	Type III	NA	NA	2.8	NA
	T	Type II	NA	NA	7	NA
	T	Type I	NA	NA	11	NA
**Zhu 2014**	T+S	Type III	10	0	3	10
	T+S	Type II	19	6	13	58
	T+S	Type I	17	7	15	82
	T	Type III	10	0	3	0
	T	Type II	21	3	6	14
	T	Type I	13	5	10	23
**Zhang 2015**	NA	NA	NA	NA	NA	NA
**Pan 2014**	T+S	Type II- III	31	NA	14.0	NA
		Type IV	10	NA	7.8	NA
**ChenJW2013**	T+S	Type III	10	0	3	20.0
	T+S	Type II	10	3	9	70.0
	T+S	Type I	12	6	14	91.7
**Wang 2016**	T+S	Type I	31	NA	12.01	NA
	T+S	Type II	45	NA	8.92	NA
	T+S	Type III	37	NA	6.96	NA
	T	Type I	47	NA	9.28	NA
	T	Type II	288	NA	4.97	NA
	T	Type III	269	NA	3.98	NA

### Adverse events

AEs were reported in all included studies and included HFSR, hemorrhage of the digestive tract, diarrhea, hypertension, fatigue, alopecia, liver dysfunction, oral ulcer, and rash/desquamation. AEs experienced during combination therapy are shown in Table 6. A total of 243 patients received combination therapy. The most common AEs were HFSR (178; 73%), diarrhea (142; 58%) and alopecia (76; 31%). AEs of grade 3/4 were rare. The studies by Chen *et al*. [21] and Luo LD and Luo Z [22] indicated that sorafenib increased the probability of HFSR and diarrhea. More AEs were associated with sorafenib in the combination groups.

**Table 6:** Adverse events

Study	HFSR (*n*/%)	Hemorrhage of digestive tract (*n*/%)	Diarrhea (*n*/%)	Hypertension (*n*/%)	Oral ulcer (*n*/%)	Fatigue (*n*/%)	Alopecia (*n*/%)	Liver dysfunction (*n*/%)	Rash/Desquamation (*n*/%)	grade3/4 adverse eventsa
**Hao 2015**	23(71.8)	1(3.1)	22(68.8)	3(9.4)	1(3.1)	NA	12(37.5)	NA	NA	4
**Chen 2014**	19(90.5)	4(19)	14(66.7)	2(9.5)	2(9.5)	NA	NA	NA	NA	NA
**Luo 2014**	19(73.08)	NA	6(23.08)	2(7.7)	9(34.61)	NA	4(15.38)	10(38.36)	NA	NA
**Zhu 2014**	37 (80)	4 (9)	33 (72)	6(13)	NA	13 (28)	15 (33)	2 (4)	NA	16
**Zhang 2015**	29(64.4)	3 (6.6)	20(44.4)	1(2.2)	NA	11(24.4)	25 (55.6)	25(55.6)	NA	16
**Pan 2014**	28(68.3)	NA	22(53.7)	4(9.8)	NA	9(22)	8(19.5)	NA	9(22)	4
**Chen JW 2013**	23(71.9)	2(6.2)	25(78.1)	3(9.4)	1(3.1)	NA	12(37.5)	NA	NA	6
**Wang 2016**	NA	NA	NA	NA	NA	NA	NA	NA	NA	NA
**total**	178(73)	14(6)	142(58)	21(9)	13(5)	33(14)	76(31)	37(15)	9(4)	NA

There was no obvious difference in AEs related to TACE between the combination therapy group and the TACE-alone group. Sorafenib did not increase the probability of AEs related to TACE. Therefore, we will not discuss differences in AEs in detail.

## DISCUSSION

Comprehensive treatments are available for HCC patients with PVTT, but high disease recurrence limits the effectiveness of these treatments. Although several studies have shown that TACE is effective in the treatment of patients with PVTT alone and the effect of TACE on improving the 1-year survival rates of patients with HCC and PVTT [27, 28], few have evaluated the results of TACE combined with other treatments applied in patients with PVTT. This study is the first systematic review and meta-analysis to aim to identify nearly all studies of documents about TACE-S for the treatment of PVTT, analyze the curative effects and the safety of this combined therapy and provide a foundation for the clinical treatment of PVTT.

In summary, the combined therapy was more effective than TACE alone. Tumor response is an important aspect of short-term curative effects. TACE-S was associated with higher ORR and DCR for patients with PVTT. With respect to the long-term curative effect, OS should be discussed, such as the median TTP, median OS and survival rate. This study revealed that combination therapy improved the 6-month and 1-year OS of HCC patients with PVTT.

Several mechanisms may underlie the complementary roles of TACE and sorafenib. Embolization of the hepatic artery by TACE reduces the blood supply of HCC, achieving the goal of treatment. However, the side effects of TACE include increased vascular endothelial growth factor (VEGF) expression, liver function damage and increased possibility of the recurrence of HCC [29]. Sorafenib is a small-molecule multikinase inhibitor with antiangiogenic properties that primarily acts through the vascular endothelial growth factor 2 (VEGFR-2) pathway, platelet-derived growth factor receptor (PDGFR) pathway and Raf signaling pathway. Sorafenib can block neoangiogenesis and HCC growth [30], significantly improving OS and TTP in patients with advanced HCC [31]. Thus, sorafenib can reduce the side effects of TACE and improve the positive effects of TACE compared to TACE alone. Recent one study has revealed collaborative efficiency of TACE using oxaliplatin and doxorubicin with sorafenib, particularly in blocking HCC growth and neoangiogenesis as well as improving OS [32]. Our systematic review included 8 trials, all of which revealed the same effect in patients with PVTT. However, only one meta-analysis [12] showed that TACE-S might be superior to TACE alone in terms of TTP but not OS in advanced HCC patients without PVTT, perhaps because these patients had no PVTT, although this finding is worth exploring further.

Regarding safety, serious AEs were rare in the 243 included patients receiving combination therapy. Only 40 patients who had serious AEs were cured. The most common AEs were HFSR and diarrhea related to sorafenib. During combination therapy, sorafenib did not increase the risks of TACE treatment [33]. Moreover, patients who had light symptoms were able to treat their discomfort, whereas patients with severe symptoms reduced the dose of sorafenib or paused their sorafenib treatment and used affordable, relevant auxiliary treatments to relieve and reverse AEs [34]. However, these methods also hamper the benefits of TACE-S. Our results were similar to those of previous meta-analyses of patients without PVTT [12–14]. Thus, the combination therapy has better tolerance and safety, and severe AEs were controllable.

Patients’ liver function and the extent of PVTT in the portal vein could determine the selection of treatments [7]. We must pay attention to the patients’ liver function and select appropriate treatments to reduce AEs. According to the study of Wang, *et al*. [7], surgery and TACE+RT may improve the overall survivals of HCC patients with PVTT. Surgery was the best treatment for type I and II PVTT patients with Child-Pugh A and selected B liver function. TACE-RT should be given to type III PVTT patients. Further, A Chinese expert consensus on multidisciplinary diagnosis and treatment of hepatocellular carcinoma with portal vein tumor thrombus (2016 edition) [35] led in our hospital has published online recently, the first expert consensus aiming to PVTT in the world, which could guide to treat these patients. Our studies concentrated on the comparison of TACE-S and TACE for PVTT. TACE-S may also improve OS of PVTT patients who could not be performed surgery and RT. This systematic review and meta-analysis may improve the level of evidences in Chinese expert consensus and provide references for PVTT patients in other countries. Finally, no RCTs were available and lack of prospective nature of studies included significantly increase the bias, and the results require confirmation in further high-quality trials.

There are several limitations of this analysis that should be considered when interpreting the findings. Although the 8 retrospective studies included in the systematic review and the 5 studies included in the meta-analysis scored 7-8, the use of different treatment options in the different studies might have influenced the reliability of the conclusions. Firstly, publication bias existed in our studies with natural quality. However, because of the limited number of included studies and hence limited statistical power, publication bias test was not performed in our article according to Begg's and Egger's recommendation [19]. Secondly, all of the included studies were conducted in China. We have systematically searched databases based on our search strategy, and all relevant studies were included to our analysis. In China, HCC is the one of the highest cancer killer and HBV infection is highly endemic. In diagnosis and treatment for PVTT, clinicians and researchers had rich experience. Thus, all the included studies designed in China have great reliability.

In conclusion, this systematic review and meta-analysis suggests that TACE-S is superior to TACE alone. It is imperative to design additional rigorous, multicenter RCTs with large samples to assess the long-term curative effects and improve the stability of TACE-S for PVTT.

## MATERIALS AND METHODS

### Search strategy

We systematically searched the Cochrane Library, PubMed, EMBASE, Chinese National Knowledge Infrastructure (CNKI), VIP, Wan Fang, and Sino Med databases, with no limitations on language and with a limitation to human studies to obtain useful data; similarly, we searched ClinicalTrials.gov to obtain available outcomes of ongoing studies. The following search strategies were used: “transcatheter arterial chemoembolization” or “TA (C)E” or “transarterial chemoembolization” or “chemoemboli*” or “emboli*” AND “sorafenib” or “Nexavar” or “Raf 1 Kinase Inhibitor II” AND “portal vein tumor thrombus” or “(portal vein thrombosis)” or “PVTT” AND “(liver or hepatic or hepatocellular or hepatocellular) and (carcinom* OR cancer OR neoplasm* OR malign* OR tumor* OR tumour*)” or “HCC” or “hepatoma*”. All abstracts were screened independently by Zhang X P and Wang M, and full-text reports of suitable papers were obtained for another screen. We also searched the relevant references of the retrieved papers.

### Study selection

Inclusion Criteria

1. HCC patients with various PVTTs.
2. Clinical trials using sorafenib plus TACE or comparing TACE-S with TACE therapy alone for the treatment of HCC patients with PVTT.
3. Trials including the following: overall survival (OS), time to progression (TTP), tumor response, and related information about the original data and that could be calculated.
4. Relevant conference summaries and degree papers about sorafenib plus TACE for PVTT, with no publication language limitation applied.

Exclusion Criteria

1. HCC patients without PVTT and patients receiving methods of treatment other than TACE and sorafenib.
2. Case reports, current affairs review, reviews and other meta-analyses and studies that did not provide all necessary information to evaluate the quality of the study.
3. For repeated publications, repetition, or information from the same study, only the latest study was used, and the others were excluded.

### Data extraction and quality assessment

All data from the trial reports were extracted and checked independently by two reviewers (Zhang XP and Wang M). If necessary, a third author (Wang K) was invited to participate in the resolution differences. Meanwhile, the reasons for excluding studies were documented.

1. The extracted basic data included the authors, year of publication, country, study design, sex of patients, number of patients, median daily dosage of sorafenib, and median number of TACE sessions.
2. Data about the state of HCC were collected, such as HCC stage (ECOG), size and characteristics of HCC (AFP), liver function (Child A/B/C), and types of PVTT.
3. Finally, some data were included about the experimental content, such as the study methods and outcomes (OS, 6month, 1 year), TTP, DCR (disease control rate), and incidence of adverse events (diarrhea, hand-foot skin reactions (HFSR), hypertension).

All data were included in the TACE alone and TACE-S groups. Three of the authors (Zhang XP, Wang M and Wang K) independently extracted the data and then entered the requisite data into RevMan software, version 5.3 (The Cochrane Collaboration. http://tech.cochrane.org, Updated June 2014). For nonrandomized, controlled trials (NRCTs), the quality of observational studies was assessed using the Newcastle-Ottawa Scale (NOS)[16] to appraise the risk of bias in the selection of study groups and comparability of groups.

### Statistical analysis

The study outcomes included OS, TTP, treatment response (CR, PR, and SD) and AEs. Tumor response (CR, PR, and SD) was evaluated using the Response Evaluation Criteria in Solid Tumors (RECIST) criteria or the modified RECIST criteria [17]. Adverse events were categorized using the National Cancer Institute criteria: PDQ^®^ Cancer Genetics Risk Assessment and Counseling. (http://cancer.gov/cancertopics/pdq/genetics/risk-assessment-and-counseling/HealthProfessional.) and National Comprehensive Cancer Network (NCCN): The NCCN Clinical Practice Guidelines in Oncology TM 2010. (www.nccn.com.). The main outcome measurements were summarized using descriptive statistics. The comparative outcomes were pooled by meta-analysis using RevMan version 5.3.

The results are presented as hazard ratios (HRs) and odds ratio (ORs) with 95% confidence intervals (CIs) for all outcomes to evaluate OS, TTP and tumor response. Some OS data were obtained from survival curves. The natural logarithm of the HR and its standard error (SE) were calculated using the relevant statistical methods and calculation sheets prepared by Matthew Sydes and Jayne Tierney, as reported previously [18]. The survival rates at different time points based on the survival curves were entered into the calculation sheet “(2a) curve data”. Accordingly, a curve was produced in the calculation sheet “(2b) curve copy”, and ln[HR] and se (ln[HR]) were available in the calculation sheet “(4) output information”. Patients who underwent at least one round of TACE-S therapy were included in the safety analysis.

Q statistics and I^2^-index, according to the suggestions of the Cochrane collaboration, were used to assess the heterogeneity of the included studies. P < 0.05 with an I^2^-index > 50% was considered to indicate significant heterogeneity. The estimates were pooled with a fixed-effect model if no significant heterogeneity was identified. Otherwise, the effects were pooled with a random-effects model that accounted for both within- and between-study variability.

### Quality of evidence and risk of bias

The quality of non-randomized studies was assessed by the NOS, which included the evaluation of risk of bias in the selection of study groups, comparability of groups, and ascertainment of the exposure or outcome of interest. Although 2 to 3 articles have published in Chinese journals, these journals, acknowledged by the peers, have a high credibility in HCC field in China. The scores ranged from 7 to 8, indicating that these studies had high quality.

Publication bias was not evaluated in any of the outcomes because the number of studies reported was less than five; thus assessing for publication bias using funnel plots, Begg's test and Egger's test was inappropriate [19].

In all sensitivity analyses, there was no significant heterogeneity or deviation among the included studies. Sensitivity analysis was performed using the leave-one-out approach in the meta-analysis, using RevMan software, version 5.3. The direction and magnitude of pooled estimates did not change when removing studies, indicating that the all meta-analyses had good reliability and were not overly influenced by one of the included studies.
